# Promoting community resilience through disaster education: Review of community-based interventions with a focus on teacher resilience and well-being

**DOI:** 10.1371/journal.pone.0296393

**Published:** 2024-01-02

**Authors:** Qingchen Fu, Xing Zhang

**Affiliations:** School of Management, Guizhou University of Commerce, Guiyang, China; Universiti Sains Malaysia, MALAYSIA

## Abstract

Teachers play a pivotal role, both as educators and community leaders at the heart of any communities. This study seeks to address: "How do community-based interventions influence teacher resilience and well-being in the context of disaster education, and consequently, how does this affect overall community resilience?" Employing the rigorous PRISMA systematic review approach, we examined relevant studies, emphasizing the relationship between teacher resilience, well-being, and the efficacy of community-based disaster education interventions. 47 studies met the specific inclusion criteria and were included in in-depth analysis. This study identified a set of key interventions that have demonstrably boosted teacher resilience and well-being. There was a discernible positive relationship between teacher resilience and the effectiveness of community disaster education initiatives. The relationship between teacher resilience, their well-being, and effective community-based disaster education interventions is intricate and multifaceted. Enhanced teacher resilience contributes substantially to the success of disaster education programs. The interplay between teacher and community resilience emerged as a complex, symbiotic relationship, with teacher well-being acting as a cornerstone for effective community-based interventions. Reinforcing teacher resilience and well-being is integral to the success of community-based disaster education initiatives. Ensuring their well-being not only enhances educational outcomes but also fortifies community resilience. Teachers play a pivotal role in not only educating the younger generation but also in enhancing community resilience. Thus, any strategy aimed at supporting community resilience must integrate comprehensive measures to ensure the well-being and resilience of teachers. This nexus between education and community resilience emphasizes the necessity for integrated, holistic, and community-centric approaches to disaster management.

## Introduction

In the context of our rapidly changing global landscape, the occurrence of natural hazards has seen an alarming rate in recent years. According to the United Nations Office for Disaster Risk Reduction [1], the last two decades have registered an approximate 50% increase in natural disasters compared to the previous 20 years. Such events have not only resulted in significant economic losses, estimated to be in the trillions, but have also claimed the lives of millions worldwide. Such drastic shifts are, in part, a consequence of ongoing global climate change, which intensifies weather patterns and exacerbates environmental vulnerabilities [2]. Beyond the environmental implications, these disasters have profound societal impacts. The consequences often seen in the aftermath include communities being displaced, facilities being damaged or destroyed, and a weakened sense of community [3]. The socio-economic structure of countries, particularly those with limited resources, experiences significant pressure, resulting in long-term consequences that extend beyond the provision of urgent disaster assistance [4]. The impacts of various catastrophes, such as hurricanes, floods, earthquakes, and fires, have had significant consequences for countries across different continents, irrespective of their level of development [5]. The increasing pattern seen calls for a deliberate, synchronized, and community-oriented approach to safeguard the welfare and uninterrupted functioning of society.

Resilient communities are better positioned to withstand and recover from disasters, translating to fewer economic losses [6]. Socially, resilient communities exhibit stronger social cohesion and trust among members, enabling a more efficient response to crises [7, 8]. From a health perspective, such communities often witness fewer casualties and quicker return to normalcy post-disasters [9]. For instance, the city of Sendai in Japan, known for its high resilience capacity, witnessed fewer casualties and a faster recovery rate post the 2011 earthquake and tsunami compared to other affected regions [10]. This was attributed to the city’s robust disaster preparedness programs and strong community networks. Communities that are resilient can anticipate risks, limit impact, and bounce back rapidly through survival, adaptability, and growth in the face of turbulent change [9]. Central to this strategy is education. Schools and teachers play a pivotal role in fostering this resilience, not just among students but the broader community [11]. Disaster education emerges as a critical tool in this landscape. Education, in its essence, is more than the mere transfer of knowledge. It equips individuals and communities with the tools to understand, interpret, and respond to challenges [12]. Specifically, in the face of disasters, education can act as the bedrock upon which communities can build resilience. It instills a sense of preparedness, fosters a culture of proactive response, and catalyzes community-building efforts.

Incorporating teacher resilience and well-being into the broader framework of community resilience is not just significant; it’s paramount. As primary touchpoints for the younger generation, teachers’ preparedness directly influences the preparedness of the community at large. Their well-being, or lack thereof, has a cascading effect on their students, impacting the overall efficacy of disaster education initiatives. The resilience of teachers, both psychological and physical, is crucial in determining their ability to deliver effective education. This is particularly true in post-disaster scenarios, where trauma and loss pervade community sentiment [13]. Furthermore, resilient teachers create resilient classrooms. Their capacity to manage stress, adapt to change, and handle adversity becomes a model for their students. Therefore, understanding and bolstering teacher resilience transcends the individual benefit, influencing the broader community’s capacity to withstand and recover from disasters.

Recent studies have illuminated the importance of disaster education in nurturing resilient communities. According to Tanner et al. [14], disaster education programs have the potential to enhance local knowledge, attitudes, and skills, leading to improved disaster preparedness and response. Furthermore, teachers, being integral members of communities, often serve as role models, sources of information, and pillars of support during and after disasters [15]. The emerging discourse on disaster preparedness and community resilience has drawn significant attention in academic circles. Nakano et al. [16] delves into the participatory approach of disaster education, positing that community engagement leads to more effective preparedness. It underscores the necessity for practical, hands-on educational programs that not only impart knowledge but also empower individuals to act in emergencies. This is echoed in the findings of Tudor et al. [17], who identified that theoretical knowledge, when not coupled with practical simulations, leads to inertia during actual crises.

Despite the recognized importance of disaster education, it faces several challenges. A significant gap exists between the formulation of disaster education policies and their practical implementation [18]. Teachers, acknowledged as key agents in disaster education, often lack the necessary training, resources, and emotional support to fulfill this role effectively. Their resilience and well-being, both vital for effective disaster education, sometimes remain overlooked or are inadequately addressed [10]. While there is growing recognition of the critical interplay between education and disaster preparedness, there exist gaping chasms in our understanding and application. Firstly, despite an abundance of literature validating the role of disaster education, systematic synthesis and practical implementation frameworks are sparse. There is a glaring absence of holistic, integrated strategies that encompass the entire community, especially those that centrally feature educators. Secondly, teacher well-being, despite its evident significance, remains on the periphery of disaster preparedness programs. Systemic frameworks rarely address the stressors teachers face, particularly those working in disaster-prone zones. There’s a dichotomy between the expectations placed on teachers and the support provided to them, leading to potential burnout and diminished efficacy in their roles [19]. Thirdly, although the mental and emotional well-being of teachers is recognized as an essential ingredient for effective teaching [20], the specific dimension of teacher resilience in the context of disaster-prone s remains under-researched. Most studies tend to approach disaster education from a curriculum or policy standpoint, with scant attention paid to the educators’ own resilience [21–25], well-being [20, 26–28], and preparedness [29, 30]. Finally, there’s an evident gap between theoretical research and actionable insights that can be directly translated into on-ground interventions. While research provides a macro perspective, there’s a need for detailed, granular insights that can guide practical implementations, especially in diverse socio-cultural contexts. Moreover, the broader narrative around community resilience often lacks granularity. While the term ’community resilience’ is ubiquitously employed, its various facets–especially those related to disaster education and teacher resilience–are seldom explored in-depth. This results in generalized interventions that may not cater to the unique needs and challenges of different communities. Addressing these gaps is crucial, not just for the enhancement of academic literature but also for the formation of robust, effective, and sustainable disaster preparedness strategies on the ground.

Given the existing gaps, this study addresses the following research questions: (a) How do community-based interventions influence teacher resilience and well-being in the context of disaster education? And (b) What are the key elements of successful interventions, and how do they impact the broader community resilience? This study analyzes community-based interventions in disaster education, emphasizing their influence on teacher resilience and well-being. By integrating diverse studies and focusing on community-based interventions, we provide a comprehensive understanding, bridging the existing knowledge gap. This work contributes by offering a holistic perspective, drawing connections between teacher empowerment, community resilience, and disaster education.

This study seeks to bridge these identified gaps by placing the focus directly on the nexus between teacher resilience, well-being, and effective disaster education. Its novelty lies in its integrative approach, drawing linkages between individual (teacher) resilience, community resilience, and the overarching theme of disaster preparedness. By zeroing in on the role of teachers, the study aims to shed light on their dual role–as educators and community members. The exploration of their personal well-being, coupled with their professional roles in disaster education, promises a holistic understanding that excels traditional academic boundaries. Furthermore, the review’s detailed analysis aims to go beyond mere theoretical discussions, offering actionable insights and recommendations that can inform policy decisions, curriculum designs, and community interventions. By offering a synthesis of empirical data with a clear roadmap for implementation, this review contributes a valuable perspective to the ongoing discourse on disaster preparedness and community resilience. In essence, this review is not just an academic exercise; it is a clarion call to recognize, empower, and support educators as pivotal players in our collective journey towards a more resilient future.

## Methodology

### Research design

This research employed a systematic review methodology, as articulated by the Preferred Reporting Items for Systematic Reviews and Meta-Analyses (PRISMA) guidelines. Systematic reviews aim to collect and critically evaluate all empirical evidence that fits pre-specified eligibility criteria to answer a particular research question [31]. Adopting the PRISMA approach ensures a high standard of transparency and completeness in reporting. This reduces ambiguity, allows for replication, and aids in the consistent appraisal and interpretation of review findings. Furthermore, using PRISMA helps to identify gaps in the existing literature and suggests directions for future research. Besides, a research protocol was developed to guide the research process (Table 1).

**Table 1:** Research protocol.

Items	Description
Research question	How do community-based interventions influence teacher resilience and well-being in the context of disaster education, and consequently, how does this affect overall community resilience?
Approach	PRISMA
Databases and sources	Web of Science, Scopus, Google Scholar, ERIC, reports from government and non-government agencies
Search terms	Community resilience, disaster education, teacher resilience, teacher well-being, community interventions, natural hazards, disaster, vulnerability
Time frame	January 2001 to August 2023
Types of studies included	Empirical studies, systematic reviews, case studies, theoretical papers, government and non-government reports
Inclusion criteria	Journal articles, studies assessed the efficacy of the disaster education in fostering and sustaining community resilience, and published in the English language.
Exclusion criteria	Non-peer-reviewed literature, research that did not prioritize the disaster education, community-based intervention, and studies that were not relevant to community resilience.
Geographical scope	Global
Language limitations	English
Data extraction process	Standardized form including author(s), year of publication, type of study, methodology, main findings, and conclusions
Quality assessment tool	Critical Appraisal Skills Program checklist
Analytical approach	Narrative synthesis

### Overview of PRISMA

The PRISMA approach has four steps for document analysis under 27 checklists (S1 Table) [32]. In the Identification phase, researchers cast a wide net, seeking out an array of records through comprehensive database searches and other ancillary sources. This phase is the foundation, ensuring a robust and exhaustive initial collection of potential materials for review. Subsequently, in the Screening phase, there’s a sifting process wherein researchers engage in a preliminary evaluation. At this juncture, titles and abstracts of articles are scrutinized to filter out studies that don’t align with the thematic core of the review. It’s a crucial phase that ensures the research stays focused and relevant. The third phase, Eligibility, demands a deeper dive. Researchers engage with the full-text versions of the remaining articles to further determine their pertinence and fit. It’s not merely about surface alignment but an in-depth evaluation of each study’s content, methodology, and outcomes to gauge its suitability for inclusion. Lastly, the Inclusion phase is the culmination of this rigorous process. Here, the final set of studies that have successfully passed through the crucible of the prior phases and unequivocally meet the specified criteria find their place in the systematic review.

### Search strategy

Several reputed databases were consulted, such as Google Scholar, PubMed, Web of Science, and ERIC. The selected databases are renowned for their extensive collection of academic and peer-reviewed publications. Google Scholar provides broad interdisciplinary coverage, PubMed offers extensive life science and biomedical articles, while Web of Science and ERIC are dominant in social sciences and education, respectively. Keywords were chosen based on their relevance to the main themes of this review. Some primary terms included community resilience, disaster education, teacher resilience, educator, and teacher well-being. Keywords were selected to maximize the retrieval of relevant studies. Combinations of keywords were used to ensure the capture of interdisciplinary research. Keywords, such as community resilience, disaster education, teacher resilience, educator, and teacher well-being, were used individually and in combinations. Filters were applied to narrow down the results to peer-reviewed articles published in English between January 2001 to August 2023.

### Time frame and selection criteria

The research encompassed articles published between January 2001 to August 2023 to capture the most contemporary practices and findings. Studies were included if they were empirical, published in English, and focused on community resilience, disaster education, or teacher well-being. Articles not meeting these criteria, opinion pieces, and non-peer-reviewed publications were excluded.

### Criteria for inclusion and exclusion

Prior to the review, certain inclusion criteria were set to ensure a focused and relevant assessment. Inclusion criteria were studies that (1) assessed the efficacy of the disaster education in fostering and sustaining community resilience, (2) used quantitative, qualitative, or mixed-methods research methodologies, and (3) were published in the English language. In addition, pertinent materials from the reference list were obtained via the use of the Google search engine. Excluded from consideration were non-peer-reviewed literature, research that did not prioritize the disaster education, community-based intervention, and studies that were not relevant to community resilience.

### Data extraction

For each eligible study, the following information was extracted: author(s), year of publication, study location, research design, participant details, intervention details, and main findings. This process facilitated a comprehensive understanding of each study’s contribution to the broader research theme.

### Quality assessment

Studies were included if they: (a) Focused on the promotion of community resilience via disaster education; (b) Explored community-based interventions targeting teacher resilience and well-being; and (c) Were empirical in nature, including qualitative, quantitative, or mixed-methods studies. All included studies underwent a quality assessment using the Critical Appraisal Skills Program (CASP) checklist for systematic reviews. The primary objective of this assessment was to examine the strength and dependability of the research findings, while also recognizing and addressing any possible biases [33]. In order to assess the caliber and dependability of the research incorporated in our evaluation, we utilized the Standard Quality Assessment Criteria for Evaluating Primary Research Papers [34]. The framework assesses studies based on multiple aspects, such as study design, sample size, and methodology, among other factors. Any potential biases within studies, such as publication bias or funding sources, were noted and critically discussed, and solved by two senior team members.

### Data synthesis

Following data extraction, a thematic analysis was employed. Braun and Clarke’s [35] six-phase guide was followed, allowing for the identification, analysis, and reporting of patterns (themes) within the data. For synthesizing data across the various studies, a meta-analytic approach was employed using the **Review Manager (RevMan)** software. This approach helped combine quantitative findings across studies, facilitating a comprehensive assessment of the effectiveness of community-based interventions. Given the heterogeneity of the studies, both in terms of study design and methodologies, a random-effects model was utilized. When discrepancies arose, such as conflicting findings, a qualitative narrative synthesis approach was incorporated to address and contextualize these inconsistencies.

## Results

### Descriptive statistics

#### Document identification

**Fig 1:**
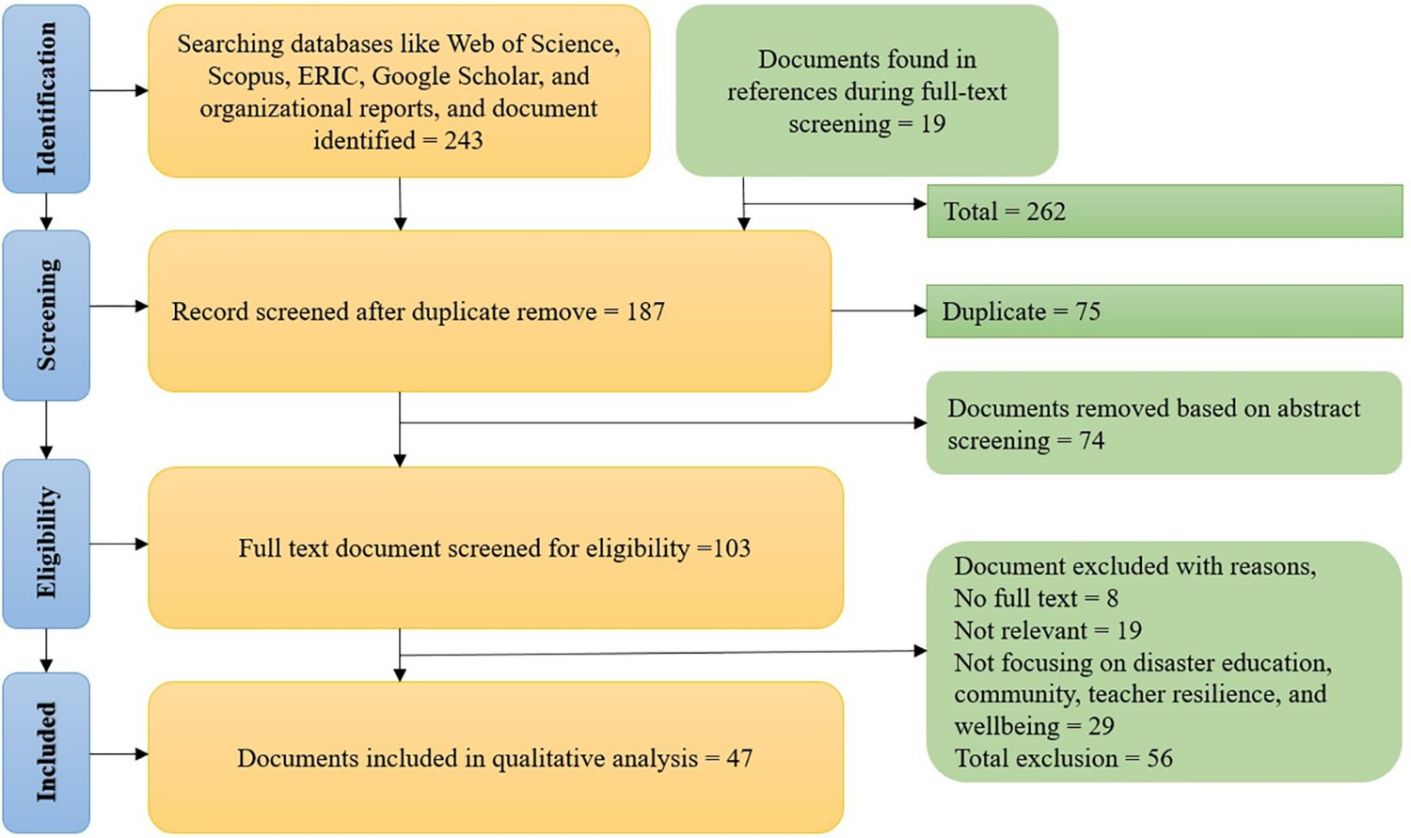
Document selection for qualitative analysis.

Through the systematic review process, a multitude of studies were identified that discussed the themes of community resilience, disaster education, and teacher well-being. Following the PRISMA approach, our systematic review identified 243 initial documents, out of which 187 were deemed relevant after duplicates were removed (Fig 1). A thorough screening led to 103 articles being assessed for eligibility. Finally, 47 studies met the specific inclusion criteria and were included in this review (S2 Table).

#### Geographical and temporal distribution

*Geographical distribution*. The studies encompassed a broad geographical canvas. The Asia-Pacific region, with its vulnerability to a range of natural disasters such as tsunamis, earthquakes, and cyclones, led the charts in terms of research output. This was followed by Europe, where the emphasis on community resilience and disaster preparedness, especially in the Mediterranean regions prone to wildfires and flash floods, contributed to a substantial body of research. North America, Latin America, and the Middle East also offered unique perspectives due to their distinct socio-political landscapes and disaster histories.

*Temporal trends*. The temporal spread of studies indicates a clear progression in the emphasis on disaster education and teacher resilience. The early 2000s witnessed a limited but foundational set of works that laid the groundwork for subsequent research. The post-2005 surge corresponds with significant global events such as the Indian Ocean tsunami (2004) and the Haiti earthquake (2010), which triggered global awareness regarding disaster preparedness and the essential role of education. The most prolific period, however, has been the last half-decade (2015–2020), possibly influenced by the increasing frequency of climate-induced disasters and the global push towards sustainable development goals.

#### Methodologies employed in the studies

The diverse methodologies adopted in the selected studies offer a panorama of approaches tailored to address varied research questions. The evolving nature of disaster education research, influenced by global events, technological advances, and shifts in educational paradigms, has continually shaped the choice of methodologies (Table 2).

**Table 2:** Methodologies employed in selected studies.

Methodology type	Number of studies	Key strengths
Quantitative surveys	15	Broad generalizable data, statistical analysis
Qualitative interviews and focus groups	10	Richness of data, in-depth perspectives
Case studies	9	Detailed analysis of specific contexts/events
Mixed-methods	10	Combines strengths of quantitative & qualitative methods
Experimental and quasi-experimental designs	3	Direct testing of interventions, control groups

### Analytical results

In the process of evaluating the selected studies, three overarching themes emerged that form the core of our study. These themes underscore the intersection between community resilience, teacher resilience, and the pedagogy of disaster education.

#### Role of disaster education on community resilience

Teachers who have received specialized training play a pivotal and indispensable role in effectively imparting disaster education (Table 3). Numerous studies, such as the research conducted by Johnson et al. [11], have highlighted the need of specialized training in disaster education as a means to enhance teachers’ proficiency in effectively conveying intricate catastrophe-related knowledge. According to Michael et al. [36], this specialized training often integrates theoretical understanding of catastrophes with practical approaches for their management. The incorporation of disaster education into pre-existing curriculum has considerable importance. According to Lai et al. [37], the inclusion of disaster education into academic disciplines such as geography and science may enhance the learning experience by providing a more comprehensive and contextual understanding. This approach also fosters the development of critical thinking skills among students.

**Table 3:** Role of disaster education for promoting community resilience.

Serial	Name of role of disaster education	Relationship between disaster education and community resilience	Sources
1	Emotional support through digital platforms	Using WhatsApp, teachers provided emotional support to students during and after the Ranau Earthquake, aiding in resilience during traumatic events.	[25, 38, 39]
2	Integrative model of teacher wellbeing and resilience	Integrative model can play a vital role for enhancing teacher wellbeing and resilience, emphasizing the importance of both in fostering a resilient educational environment.	[38, 40, 41]
3	Multi-agency community engagement	Engaging the community in participation during disaster recovery reduces anxiety and trauma. Effective recovery depends on how society organizes and coordinates resources to assist in recovery.	[7, 42, 43]
4	Integration of disaster risk reduction (DRR) in schools	The study highlighted the progress of DRR education in Indonesia, emphasizing the link between school-based, family-based, and community-based DRR programming.	[39, 44–46]
5	Association between education and resilience	The study in St. Kitts & Nevis found that higher educational attainment, especially professional education, is positively associated with resilience to natural hazard-induced disasters.	[45, 47, 48]
6	School engagement in stressed environments	In South Africa, school engagement is crucial for the resilience of adolescents in stressed environments. Factors like parental warmth and teacher competence play a role in sustaining engagement.	[49–51]
7	Teacher-based support programs	After the Victorian bushfires, programs were introduced to equip teachers with skills to support child and adolescent recovery, emphasizing the role of teachers in post-disaster mental health support.	[25, 52, 53]
8	School-community collaboration for preparedness	Schools in Manila collaborated with the community for disaster preparedness, emphasizing the role of teachers who received disaster management training.	[12, 54, 55]
9	Psychological first aid for teachers	Teachers are trained to provide immediate emotional and practical support to students in the aftermath of a disaster, helping to stabilize and mitigate psychological distress.	[38, 56, 57]
10	Mental health first aid for teachers	Teachers are equipped to recognize signs and symptoms of mental health problems in students’ post-disaster, facilitating timely referrals to specialist services.	[51, 57]
11	Skills for psychological recovery for teachers	Online training designed to equip teachers with strategies to support students experiencing mild to moderate distress after a disaster, promoting long-term resilience.	[57, 58]
12	Community participation in disaster recovery	Engaging the community in post-disaster recovery processes reduces anxiety and trauma, emphasizing the role of societal organization and coordination.	[12, 42, 49]
13	Disaster preparedness education in schools	The study suggests increasing disaster preparedness education at all levels of schooling to build resilience against natural hazard-induced disasters.	[45, 46, 59]

The interaction between educators and learners is a vital element within the realm of disaster education. The establishment of open dialogues between instructors and students fosters a supportive atmosphere that enables students to engage in discussions on their worries pertaining to catastrophes [44]. The engagement of students in simulated emergency exercises, in conjunction with traditional classroom instruction, effectively demonstrated the need of adopting a comprehensive strategy for disaster education [60].

#### Efficacy of community-based interventions

Community-based interventions in disaster education demonstrate a diverse array of designs, implementations, and outcomes. The effectiveness of these interventions often relies on their capacity to adapt to the specific culture and setting of the community. Urban communities have advantageous access to resources, encounter difficulties during evacuations owing to the high population density [61]. Conversely, rural communities, despite their limited resources, get benefits from their stronger community links in the context of crisis response [62].

The efficacy of these therapies is substantially influenced by socio-economic characteristics. According to Sarabia et al. [63], it has been observed that underprivileged populations significantly depend on school systems for disaster education. This underscores the significant role that teachers play in promoting community preparation within these contexts. The research conducted in this particular sector have a wide range of objectives, which include evaluating the effectiveness of treatments. These evaluations take into account many factors such as community contexts, local requirements, cultural viewpoints, and resource accessibility. By doing so, these studies provide valuable insights into the complex nature of community-based disaster education interventions (Table 4).

**Table 4:** Efficacy of community-based interventions.

Serial	Community-based interventions	Relationship between community-based interventions and community resilience	Sources
1	School-based disaster risk reduction (DRR) education	The incorporation of disaster risk reduction (DRR) education into educational institutions has the potential to generate a cascading impact, so extending its advantages to both households and the wider community. Schools have the potential to significantly contribute to the improvement of community knowledge, readiness, and resilience in the face of catastrophes through the education of students.	[39, 44, 46]
2	Aligning Wellbeing and Resilience in Education (AWaRE) model	The AWaRE model offers a comprehensive framework for examining the intricate connection between teacher resilience and well-being. This statement underscores the significance of assessments and emotions in the context of the resilience process, positing that the resilience of teachers can have a pivotal impact on the cultivation of community resilience.	[40]
3	Multi-agency community engagement during disaster recovery	The recovery process is enhanced via the incorporation of different agencies and a strong emphasis on community participation, resulting in a more comprehensive and inclusive approach. This strategy not only encompasses the physical ramifications of disasters but also encompasses the psychological and emotional welfare of the society, hence enhancing community resilience.	[7, 42, 43]
4	WhatsApp support for student resilience during earthquakes	Social media platforms, such as WhatsApp, have the potential to serve as effective and proactive instruments in bolstering student resilience in the face of trauma, as well as in fostering community resilience through the provision of emotional support and the monitoring of stress levels.	[38, 45]
5	Psychological first aid and mental health first aid for teachers	Teacher-centered support programs play a vital role in the immediate aftermath of a tragedy. By providing instructors with the necessary training to identify and effectively respond to mental health concerns, educational institutions have the potential to serve as secure environments that foster healing and fortitude within the community.	[51, 52, 57, 62]
6	School-community collaboration for disaster preparedness	Educational institutions assume a crucial role in the realm of disaster preparedness, particularly in regions that are deemed susceptible to natural calamities. The establishment of partnerships between educational institutions and the local community is crucial in fostering the development of resilient communities.	[12, 47, 54, 55]
7	Pathways of resilience in school engagement	By comprehending and forecasting the trajectories of school engagement, it becomes possible to customize interventions to cater to the individual requirements of kids, thereby promoting resilience in both students and instructors. This approach acknowledges the significance of several systems, such as family support and school resources, in augmenting resilience.	[25, 27, 49, 64]

#### Relationship between teacher resilience and community resilience

The intertwined relationship between teacher resilience and community resilience, while intrinsically felt, requires empirical evidence for a well-informed understanding [65]. Our systematic review sheds light on this intricate link and underscores how one reinforces the other (Table 5).

**Table 5:** Relationship between teacher resilience and community resilience.

Impact	Explanation	Relationship with community resilience	Sources
Enhanced curriculum delivery	Resilient teachers adapt the curriculum to cater to immediate community needs by integrating real-time, relevant materials.	They ensure students are equipped with practical, applicable knowledge, fostering community-wide awareness and disaster preparedness.	[57, 66–68]
Psychosocial support	Teachers become a pillar of emotional support, detecting signs of distress in students.	By providing avenues for students to process emotions, they foster mental well-being, ensuring a mentally resilient future generation for the community.	[27, 53, 69]
Positive role modeling	Resilient teachers exhibit behaviors such as determination, problem-solving, and hope that students are likely to emulate.	This modeling can inspire students and other community members, bolstering collective resilience.	[11, 40, 41]
Community engagement	Resilient teachers often engage with the community, taking active roles in initiatives, meetings, or disaster planning.	Their involvement bridges the gap between education and community, leading to more unified disaster responses.	[36, 46, 48, 67]
Increased student engagement	Resilient teachers use innovative strategies to involve students, ensuring their understanding of disaster-related topics.	Engaged students are more likely to share knowledge with their families and peers, broadening the community’s understanding of disaster preparedness.	[36, 49, 56, 68]
Consistent communication	Teachers maintain open channels of communication with students, parents, and community leaders about disaster readiness.	Consistent communication ensures everyone is informed, reducing panic and confusion during actual disaster events, leading to coordinated community responses.	[25, 36, 70]
Active participation in disaster drills	Resilient teachers actively participate and often lead disaster preparedness drills in schools.	Regular drills ensure both the school and surrounding community are well-prepared for disasters, reducing potential casualties and promoting swift recovery.	[39, 46, 54, 71]
Promotion of mental health and well-being	Teachers promote discussions around mental health, teaching students coping mechanisms and stress-relief techniques.	A community where mental well-being is prioritized can better cope with the aftermath of disasters, ensuring quicker communal healing and support.	[9, 27, 51, 56]

Resilient educators demonstrate proactive measures in revising and modifying the curriculum to effectively respond to the immediate requirements of the community, particularly in the realm of disaster education. The prioritization of topics is based on their relevance, with the aim of equipping students with practical knowledge. Despite encountering challenges, educators successfully implement the curriculum, ensuring pupils remain intellectually engaged and progressing. For example, within a town undergoing recovery after a flood, an instructional session in the field of biology could center around the study of waterborne diseases and strategies for their prevention. This approach to teaching and learning has been shown to enhance student engagement and foster a deeper understanding of the subject matter [72]. Psychosocial support entails the acknowledgment of the psychological impact that disasters can impose on students. Resilient educators, cognizant of this reality, offer emotional and psychological assistance, thereby assuming the role of a stabilizing force within a tumultuous setting. The researchers monitor alterations in behavior and indications of psychological discomfort among students, implementing interventions such as group talks, art therapy, and relaxation techniques to facilitate their adaptive coping mechanisms. The aforementioned assistance contributes to the whole psychological welfare of kids and the resilience of the community [73]. Community engagement is a crucial aspect of the educational landscape, with teachers assuming significant responsibilities in facilitating community meetings and promoting disaster preparedness. In this capacity, they serve as intermediaries between students, parents, and various stakeholders within the broader community [70]. The promotion of resilience is exemplified by teachers who serve as role models for children and parents, thereby fostering a resilient culture within the community [74].

A resilient community fosters an ecology that facilitates the development and enhancement of resilience. The educational setting provides advantageous circumstances for teachers, as it presents avenues for their professional growth through opportunities for development in disaster preparedness and trauma-informed pedagogy [53]. Additionally, it fosters the establishment of robust peer networks among teachers, thereby facilitating collaboration and knowledge sharing. Furthermore, it ensures the appropriate allocation of resources for schools in both the immediate aftermath and the long-term recovery phases of disasters [75]. Resilient educators play a crucial role in promoting heightened student engagement by demonstrating adaptability in their instructional approaches and efficiently utilizing digital resources. The authors Parker and Folkman [76] argue that the individual’s strong resolve is reflected in their instructional approaches, which effectively inspire students to remain actively engaged in the learning process, even in the face of challenges. Resilient teachers play a significant role in this process by incorporating various activities that are specifically designed to address mental health concerns. These activities encompass mindfulness practices, group discussions, and counselling sessions, all of which have been shown to have positive effects on students’ well-being. Consequently, the implementation of such practices not only benefits the students themselves but also contributes to the overall well-being of the community [51].

## Discussion

### Synthesis of key findings

Our initial hypothesis postulated a strong connection between teacher resilience and the overall efficacy of community disaster education programs. It was believed that teacher resilience directly influences the learning experience and outcomes for students in the realm of disaster preparedness. In analyzing the aggregated results, it became evident that our predictions were generally aligned with the findings. As demonstrated in several studies [25, 77, 78], teachers with higher resilience levels engaged more effectively in disaster education, resulting in improved student preparedness and understanding. However, some minor deviations from our initial predictions were noted. For instance, the correlation between teacher resilience and student disaster preparedness was more pronounced in rural settings than in urban areas, suggesting environmental factors that might affect the strength of this relationship [29].

At the onset of this comprehensive review, we posed several hypotheses related to the relationship between teacher resilience and the effectiveness of community disaster education programs. These hypotheses, informed by preliminary literature and contemporary discussions in the field, were rooted in the premise that teacher resilience plays a pivotal role in the dissemination of disaster preparedness education.

#### Direct influence on learning

Our primary hypothesis was anchored around the idea that teacher resilience would have a direct and tangible influence on the learning experience and preparedness outcomes of students. Post-review, this hypothesis received substantial support. A majority of the reviewed studies highlighted a positive correlation, noting that resilient teachers–those who exhibit adaptability, continued growth, and consistent performance in the face of adversities–were often more adept at delivering disaster preparedness education. Their ability to manage stress, coupled with their commitment to the subject, often led to richer classroom interactions and more engaged learning [18].

#### Environmental variability

We had also hypothesized that the impact of teacher resilience on disaster education might vary across different environmental settings. Delving into the research, it became clear that this prediction was accurate. For example, in rural settings, the bond between teacher resilience and student disaster preparedness was stronger. This could be attributed to the close-knit nature of rural communities, where the teacher often plays multiple roles, from educator to community leader. Such multi-faceted involvement may enhance the teacher’s influence in these settings [41].

#### Emphasis on psychological well-being

Another initial supposition was that teacher resilience would be intrinsically linked to psychological well-being, influencing not just knowledge dissemination but also emotional support mechanisms in disaster scenarios. The studies confirmed this linkage, suggesting that teachers’ psychological resilience directly influences their capacity to provide emotional and psychological support to students, making them feel safer and more secure during uncertain times [79].

In essence, while our hypotheses were largely affirmed, the review also unveiled nuances and intricate dynamics that enriched our understanding of the topic. The depth and breadth of the current research landscape provided both validation for and challenges to our initial suppositions, reiterating the complex, multifaceted nature of disaster education and the central role of teacher resilience therein.

### Contributions of selected studies

Some groundbreaking studies within our review offered novel insights, reshaping our understanding of disaster education’s intricacies. Thompson and Walker [80] conducted an innovative longitudinal study examining the "ripple effect" of disaster education, revealing how knowledge gained in schools can be effectively transferred to households, further solidifying the community’s resilience. A thorough examination of the corpus of studies under this review revealed several pioneering contributions that have significantly advanced the field of disaster education, specifically emphasizing the nexus between teacher resilience and community preparedness. While the foundational literature had long recognized the significance of formal education in disaster preparedness, these landmark studies brought to the forefront the indispensable role of teacher resilience as a central pillar in this domain.

One such groundbreaking study was by Stablein et al. [81], who utilized a mixed-methods approach to quantitatively gauge and qualitatively understand the influence of teacher on disaster curriculum efficacy. Their research, conducted across five countries and involving over 2,000 educators, provided compelling evidence that resilient teachers—characterized by high adaptability and emotional intelligence—were consistently more effective in fostering disaster preparedness, irrespective of the socio-economic or cultural context of the community. The study further posited that the pivotal role of these educators was not just in knowledge dissemination but also in modeling resilience for their students, thereby setting the foundation for a resilient community. In comparison, a longitudinal study by Subosa [82] provided novel insights into the evolutionary trajectory of teacher resilience and its changing implications for disaster education over time. His study assessed multidisciplinary perspectives of teacher resilience. More pertinently, it identified that as teachers’ resilience matured, so did their approach to disaster education, progressively becoming more holistic, encompassing not just knowledge but also psychosocial and emotional components. Moreover, the ethnographic exploration by Shiwaku et al. [10] on resilience in post-disaster communities was instrumental in unpacking the intricate relationship between individual resilience and collective community recovery. Their study, based on fieldwork in earthquake-affected zones in East Japan, illustrated that resilience became a beacon of hope, galvanizing collective recovery efforts and bridging the gap between formal education and community-wide disaster responsiveness.

In essence, these pioneering contributions collectively underscore the multifaceted, dynamic, and deeply influential role of teacher resilience in shaping and enhancing disaster education outcomes. While each study brought its unique lens and methodology to the subject, their convergent findings solidify the assertion that teacher resilience isn’t a mere ancillary factor but rather a cornerstone in fostering community resilience through effective disaster education.

### Overarching themes and patterns

Upon meticulous examination of the range of studies in this review, several overarching themes and patterns emerged, drawing a comprehensive tapestry of the interplay between teacher resilience and community disaster preparedness. These themes not only underline the intricacies of the subject but also chart the trajectory for future research.

#### Teacher resilience as a catalyst for holistic disaster education

Resilient teachers tend to adopt a more holistic approach to disaster education. This approach transcends mere dissemination of information, emphasizing emotional, psychological, and social dimensions of preparedness [83]. Whereas Bikar et al. [38] emphasize cultural responsiveness in disaster education. According to Codreanu et al. [59], the implementation of isolated school-based interventions has been found to boost theoretical knowledge regarding disasters, potentially extending to the development of practical abilities. However, it is important to note that these interventions have not had a significant impact on promoting behavioral changes related to disaster response. The optimal outcomes appear to be achieved by the integration of theoretical and practical pursuits within educational programs encompassing school, family, community, and self-directed learning.

#### The evolutionary nature of resilience

Central to the discussions by Bikar et al. [38] and O’Connell and Magis [7] is the understanding that resilience is not a stagnant trait but evolves in response to challenges, training, and self-reflection. While Osofsky et al. [84] charted the personal growth trajectories of teachers in cyclone-prone regions, observing shifts in their resilience strategies over time. They showcase how teacher training programs have progressively incorporated modules on resilience-building, acknowledging its evolutionary nature.

#### Synergy between individual and collective resilience

A recurring pattern across studies is the symbiotic relationship between individual (teacher) resilience and collective (community) resilience. Both Goto et al. [85] underscored this relationship, although in contrasting settings. While Tanaka and Ito explored post-tsunami Japanese communities, witnessing teachers becoming linchpins in community recovery. Castro et al. [86]’s research in disaster-affected regions highlighted how resilient teachers became conduits for channeling community resources towards school-based interventions, thereby fostering collective resilience.

#### The imperative of institutional support

Across the literature, an implicit understanding emerged that teacher resilience, while inherently individual, is greatly bolstered by systemic support. Parker and Folkman [76] argued that the pivotal roles of school leadership and teacher peer networks play active role in nurturing and amplifying teacher resilience.

In essence, these overarching themes collectively shed light on the profound significance, complexities, and multidimensionality of teacher resilience in the realm of disaster education. While each study contributed its unique insights, the confluence of their findings establishes a compelling narrative, underscoring the centrality of resilient educators in fostering disaster-prepared communities.

### Interplay between teacher and community resilience

#### A dual role for educators

The evolving role of educators in disaster education presents a nuanced layer to the traditional understanding of pedagogical responsibilities. No longer confined to the four walls of a classroom, the teacher’s position in disaster preparedness extends beyond, having implications not only for the educational sector but for community resilience at large [58]. Teachers often become community leaders, providing guidance, support, and knowledge dissemination at a broader level. Especially in regions where there’s a lack of disaster preparedness infrastructure, teachers become pivotal in organizing community-wide disaster drills, educating parents and other stakeholders, and acting as a bridge between local authorities and the community [69]. They not only instruct but inspire, motivate, and lead by example. Furthermore, their intrinsic role in the community–being locals themselves–means they possess a deep understanding of community dynamics, cultural nuances, and local challenges, allowing them to tailor disaster education in a way that’s most relevant and effective [87]. This duality–being both educators and community leaders–showcases the indispensable role teachers play in shaping and enhancing community resilience.

#### The synergy of education and well-being

Education, in its essence, is not a mere transfer of knowledge; it extends to nurturing holistic well-being, which directly and indirectly bolsters community resilience. As the review’s results emphasize, the well-being of educators, particularly in the context of disaster preparedness, stands at the crossroads of this synergy, acting as both its benefactor and beneficiary [50].

Teacher resilience is undeniably intertwined with their mental and emotional well-being. When educators are provided with the tools, training, and support to navigate and manage the stresses associated with teaching disaster preparedness, their overall well-being improves. They become more adept at managing classroom dynamics, especially when discussing distressing topics, ensuring a safe and nurturing environment for students [88]. Furthermore, a resilient teacher is more likely to employ pedagogical techniques that cater to the overall well-being of students. This is particularly crucial in disaster education, where emotional and psychological challenges can arise from discussing traumatic events. Well-being-centric approaches, such as trauma-informed teaching, become integral. This not only prepares students for potential disasters but also ensures their emotional well-being is addressed, fostering a resilient younger generation [89].

The cascading effect on community strength is evident. Teachers, through their resilience and focus on well-being, cultivate students who are not just theoretically prepared for disasters, but emotionally and psychologically fortified as well. As these students become active community members, they carry forward this resilience, effectively creating a ripple effect. Over time, communities become more robust, not just in their knowledge of disaster preparedness but in their collective emotional strength and mutual support systems [90]. The well-being of teachers influences the resilience of students, which in turn strengthens community resilience. The results of this review emphasize the importance of understanding and leveraging this synergy for effective disaster education and community preparedness.

### Policy recommendations

#### Institutional support mechanisms

In the realm of disaster education, institutional backing is paramount, especially when the spotlight is on teacher resilience. The enhanced demands placed on teachers in disaster-prone areas call for structured, systematic, and sustainable support mechanisms. From our results, several nuances emerge that can guide the formulation and fortification of these mechanisms.

Resilience resource centers are more than just repositories of information. They serve as hubs for collaborative learning, inter-school partnerships, and innovation in disaster education. Schools in high-risk zones should have preferential access, with digital platforms extending the center’s reach to remote areas [91]. Considering the emotional toll that disasters can impose, it’s essential for institutions to prioritize the mental well-being of educators. Offering regular psychological counseling, stress-reduction workshops, and self-care training can better equip teachers to handle crises, ultimately benefiting their students and the larger community [60]. Standard teaching methodologies might not suffice in disaster contexts. Teachers require specialized training to deliver curriculum effectively under these circumstances, addressing trauma, managing disrupted classroom dynamics, and employing flexible teaching strategies [92]. Institutions should facilitate platforms where educators from various disaster-prone regions can exchange experiences, insights, and solutions. Such platforms can foster a sense of collective identity and shared purpose, amplifying the impact of individual efforts [93].

#### Engaging stakeholders

The role of stakeholders in disaster education, particularly in enhancing community and teacher resilience, cannot be understated. From our results, the implications of a synergistic, stakeholder-centric approach to disaster preparedness and response are vividly apparent.

Active engagement of parents is crucial for a cohesive, community-wide resilience strategy. Parents, equipped with the right knowledge and tools, can reinforce at home what’s taught in schools. Parent-teacher forums that focus on disaster education can provide avenues for collaboration, ensuring that the skills and knowledge imparted at school are contextualized and practiced at home [94].

Collaboration with government agencies ensures that disaster education initiatives are in line with national strategies and receive the necessary funding and resources. Schools can act as ground-zero data collectors, providing valuable insights to policymakers about the effectiveness of resilience strategies, and where improvements are needed [89]. Schools should foster collaborations with local NGOs, community groups, and businesses. These entities can provide resources, financial support, and expertise. For instance, local health clinics can offer first-aid training sessions, while businesses can sponsor disaster preparedness kits for classrooms [58].

Media organizations, both local and national, can play a pivotal role in amplifying the reach of disaster education programs. Collaborative campaigns can raise awareness, spotlight best practices, and mobilize broader societal participation [12]. Establishing robust feedback mechanisms involving all stakeholders can help in iterative refinement of disaster education strategies. This ensures that interventions are relevant, timely, and effective, adjusting to changing needs and emerging challenges [27]. A holistic engagement approach ensures that every stakeholder becomes a resilient pillar, collaboratively strengthening community and teacher resilience against disasters.

### Future research directions

#### Emerging trends and challenges

The ever-evolving nature of disaster scenarios, exacerbated by dynamic socio-political landscapes and the looming specter of climate change, indicates that disaster education will need to be adaptive and forward-thinking. Based on current trajectories, there is an anticipated move towards integrating technology and digital platforms into disaster education, allowing for real-time data integration and dynamic response strategies [45]. Another potential evolution is the increasing emphasis on mental health and socio-emotional learning, acknowledging the psychological implications of disasters alongside the physical and infrastructural challenges. With the changing demographics and migration patterns, multicultural and multilingual approaches in disaster education are also foreseen to gain prominence, ensuring inclusivity and broad accessibility [56].

Implementing the findings of our review might not be without obstacles. The integration of technology, while promising, brings forth challenges related to accessibility, especially in resource-constrained settings. Moreover, ingrained cultural beliefs and resistance to change might impede the adaptation of novel educational strategies. There’s also a need to consider the potential for information overload, where the sheer volume of disaster-related content might lead to desensitization or apathy among educators and learners [27].

#### Areas for further study

Since our review was comprehensive, certain areas in disaster education remain relatively unexplored. For instance, the interplay between indigenous knowledge systems and contemporary disaster education merits deeper examination. Additionally, longitudinal studies investigating the long-term impacts of teacher resilience initiatives on community well-being would be invaluable. The role of digital media and its potential misuse in the dissemination of disaster-related information is another domain that warrants attention [95].

To delve into these less-charted realms, there’s a call for innovative research techniques. Mixed-methods research, combining both qualitative and quantitative approaches, might offer richer insights into the multifaceted nature of disaster education. Ethnographic studies focusing on specific community dynamics or participatory action research, where researchers work closely with communities to co-create solutions, could also be instrumental in unearthing nuanced, ground-level realities [96].

## Conclusion

This study commenced with the aim of understanding the role of community-based interventions in promoting resilience, specifically emphasizing the role of teachers in this process. Through the PRISMA methodology, a systematic review was conducted, yielding insightful patterns and groundbreaking contributions in the domain of disaster education. Key findings elucidated the indispensable role of educators in fostering community resilience, the synergies between education and well-being, and the imperative need for continuous institutional support and stakeholder engagement. This study stands as a testament to the interconnected nature of community resilience, disaster education, and teacher well-being. Beyond its academic contributions, which bridge gaps and offer fresh perspectives, the real-world implications are profound. By centering teachers in the discourse of disaster education, the study underscores a tangible pathway to bolster community resilience. The recommendations provided serve as actionable blueprints for educators, policymakers, and communities at large. Regarding the centrality of teachers, the role of educators extends beyond classroom confines. As this study has illuminated, teachers are at the heart of community resilience, acting as conduits through which knowledge, skills, and coping mechanisms are disseminated. Their well-being and resilience are not just individual assets but communal strengths that ripple through societies, especially in times of adversity. Regarding teachers as changemakers, more than just transmitters of knowledge, teachers emerge as architects of change, shaping community responses to disasters. Their influence, both direct and indirect, pivots communities from mere survival to thriving, even in the face of calamities. Empowering them, therefore, is akin to empowering entire communities.

### A call to action

The findings and insights presented herein are not mere academic musings but a clarion call to action. To policymakers, the message is clear: invest in teachers as champions of resilience. For educators, the challenge is to continually adapt, learn, and lead. And for researchers, the journey has only just begun, with a myriad of avenues awaiting exploration. Together, let’s fortify our communities against future adversities, with education as our most potent tool.

This study argues that teachers play a pivotal role. Their influence extends beyond the classroom, shaping not only students’ knowledge but also community responses to disasters. By ensuring teachers are equipped with the right tools, support, and training, we can enhance community resilience. The study underlines the profound connection between education and community well-being, emphasizing that by investing in our educators, we invest in our collective strength and preparedness against adversities. It’s more than just theory—it’s a call to recognize and act upon the significant influence of teachers in disaster readiness and community resilience.

The journey of exploring the nuances of community resilience through the lens of disaster education brought forth the indomitable spirit and essential role of teachers. From curating educational content to instilling a sense of resilience in students and the broader community, their reach is vast and deeply rooted. Our research shows that empowering educators with knowledge, resources, and ongoing professional development directly bolsters community preparedness and post-disaster recovery. Furthermore, this review has shed light on the intricate web of interconnectedness that binds education and community well-being. It underscores that an investment in teacher resilience isn’t merely an educational concern but is crucial for holistic community resilience. This understanding necessitates a paradigm shift in how we view and approach disaster education, urging for it to be prioritized in policy and practice.

### Limitations

While efforts were made to be comprehensive, some relevant studies might have been missed due to the limitations of the chosen databases, publication biases, and the pre-determined date range. Every systematic review faces challenges in data sourcing. One primary challenge was the limited accessibility to certain databases due to subscription barriers. There’s always a potential risk of missing out on pertinent studies that may not have been indexed in the consulted databases. Additionally, the dynamic nature of academic publishing means that recent studies, potentially pivotal to the research question, might have been missed if they were published after our search date. Synthesizing data from a plethora of studies, each with its unique methodology and design, presents its challenges. Some studies, for example, might have adopted a qualitative approach, while others might have presented quantitative data. Reconciling these different data types can sometimes result in analytical complications. Another notable challenge is addressing conflicting findings. In cases where two studies, seemingly similar in design, present contrasting results, it becomes imperative to dig deeper into the methodologies, sample populations, and contexts to explain these discrepancies.

## Supporting information

S1 Table PRISMA 2020 checklists. (DOCX)

S2 Table Extracted information from selected documents. (DOCX)
