# Supplementary material for: Promoting community resilience through disaster education: Review of community-based interventions with a focus on teacher resilience and well-being
Source: PLoS One. 2024 Jan 2;19(1):e0296393. doi: 10.1371/journal.pone.0296393 (PMC10760850; doi:10.1371/journal.pone.0296393)
Supplement: S2 Table — (DOCX) [file pone.0296393.s002.docx]

**Table S2**. Extracted information from the selected documents

| Serial | Author(s) | Publication time | Objective | Methodology | Key Findings | Implications |
| --- | --- | --- | --- | --- | --- | --- |
| 1 | Kangas-Dick  et al. [1] | 2020 | Discuss teacher resilience and its role in preventing attrition and burnout. | Systematic review of literature. | Interventions targeting contextual factors and integrating resilience programs at the school level are most effective. | The establishment of secure and cooperative environments for both teachers and students hold significant importance. |
| 2 | Johnson et al. [2] | 2016 | Evaluate disaster education programs for children. | Review of frameworks for program theory development. | Emphasizes the need for an explicit program theory for effective disaster education. | Importance of theory-based approaches in disaster education evaluation. |
| 3 | Cutter et al. [3] | 2008 | Understand and measure community resilience to disasters. | Development of the DROP model. | Introduced the DROP model for comparative assessments of disaster resilience. | The necessity of establishing standards and metrics for assessing disaster resilience within communities is evident. |
| 4 | Apronti et al. [4] | 2015 | Explore the presence and nature of DRR in Ghana's basic school system. | Content analysis and survey. | Identified gaps between disaster pedagogy in theory and practice. | Need for teacher training and appropriate learning materials for effective DRR education. |
| 5 | Mead et al. [5] | 2021 | Discuss the role of a CoP in supporting faculty during the COVID-19 crisis. | Case studies from faculty members. | Demonstrated the importance of an emergent learning framework in a CoP during a crisis. | Importance of CoP in supporting faculty during significant changes and crises. |
| 6 | Zhu and Zhang [6] | 2017 | Explore the outcome of disaster education in Chinese schools post-Wenchuan earthquake. | Survey based on students' and teachers' perspectives. | Found overall positive outcomes of disaster education, but also identified areas of improvement. | Emphasizes the need for enhanced school-based disaster education. |
| 7 | Edmeade and Buzinde [7] | 2021 | Examine the various adaptive methods that educators utilize in order to cultivate their personal resilience. | In-depth interviews with teachers. | Identified reliance on social networks and positive narratives as key adaptive strategies. | Highlights the holistic needs of teachers and their role in community resilience. |
| 8 | Wiwik Astuti et al. [8] | 2021 | Investigate how recent disasters affect teachers' knowledge, attitudes, and perceptions of DRR. | Data from 97 teachers in six schools. | Found that disaster experience improved teachers' knowledge about DRR. | Emphasizes the importance of integrating disaster experiences into DRR education. |
| 9 | Oktari et al. [9] | 2018 | This study investigates the current state of the school-community collaboration network in Aceh and its effectiveness in building disaster resilience. | Case study; Interviews, FGDs, survey | Schools and other stakeholders gain from school-community collaboration networks. | Highlights the value of a robust network of collaboration between the community and schools in order to improve community resilience. |
| 10 | Dufty [10] | 2009 | Boost the efficacy of Australia's school natural hazard programs. | Survey | Some school natural hazard programs might not be as effective as they could be. | Offers recommendations on how to make school natural hazard programs more successful. |
| 11 | Seyle et al. [11] | 2013 | Analyze how teacher effectiveness perceptions and classroom conduct are related to post-earthquake suffering. | Interviews, intervention design, and assessment. | Negative classroom behavior is significantly correlated with instructors' melancholy. Treatment for posttraumatic stress disorder and depression was effective. | Educators assume a vital function in facilitating pupils' ability to manage persistent stress within their local environment. |
| 12 | Codreanu et al. [12] | 2014 | Determine whether current approaches to disaster education for adolescents improve their understanding and adaptability. | Systematic literature review. | Awareness of theoretical disasters is improved by isolated school-based interventions, but no change in behavior is observed. | Changing behavior regarding disaster preparedness requires a concerted educational effort. |
| 13 | Sung-Chin Chung and Cherng-Jyh [13] | 2016 | Examine the level of expertise in disaster prevention possessed by Taiwanese school administrators and educators. | Quantitative surveys | The level of disaster prevention knowledge among the participants was the lowest, while disaster prevention talents were in the highest. | Emphasizes the importance of comprehensive disaster prevention education. |
| 14 | Gaillard and Mercer [14] | 2013 | Discuss the knowledge-to-action divide in the context of disaster risk reduction (DRR). | Literature review and analysis. | Local and scientific expertise, as well as bottom-up and top-down actions, are required for an integrative process. | Proposes ways to link gaps in DRR between various actions and knowledge sources. |
| 15 | Cels et al. [15] | 2023 | Determine the level of preparedness for tsunamis within the education sector of Sri Lanka. | Semi-structured interviews. | Schools rely heavily on tsunami early warning towers, but many are not within audible range. Low level of baseline hazard preparedness. | Need for alternative early warning channels and regular school practice drills. |
| 16 | Jarupongputtana et al. [16] | 2022 | Integrate understanding of digital citizenship competencies with principles of interdisciplinary community-based learning. | Content analysis based on grounded theory. | Distinguished concepts of interdisciplinary community-based learning and a variety of digital competencies that foster digital citizenship. | The promotion of digital citizenship is significantly aided by community-based learning. |
| 17 | Johnson et al. [17] | 2014 | Evaluate how disaster preparedness education is being implemented on a national level in primary schools across New Zealand. | Focus group study and nationally representative survey. | Factors that encourage, hinder, or promote resource utilization have been identified. | Recommendations provided for increasing use of the resource and integrating disaster education into the curriculum. |
| 18 | Abrash Walton et al. [18] | 2021 | Assess evidence-based interventions and metrics while analyzing community resilience initiatives in the Northeastern United States. | Systematic literature review. | Profound health outcomes associated with community resilience have been attributed to identified effective interventions. | Community resilience building initiatives suggested include the COAST project, COPEWELL Rubric, and Ready CDC intervention. |
| 19 | Thayaparan et al. [19] | 2014 | Dialogue concerning the challenges that higher education institutions (HEIs) encounter while providing disaster management education. | Quantitative surveys | Emphasizes the importance of lifelong learning and collaboration with external organizations for effective disaster management education. | Highlights the need for higher education-industry collaboration to address challenges in disaster management education. |
| 20 | Michael et al. [20] | 2007 | Examine family and community involvement in schools and its impact on academic achievement and school programs. | Surveys and interviews. | Family and community involvement in schools is linked to academic achievement improvements, better attendance, and improved school programs. | Suggests that many schools are not maximizing family involvement and that improvements can support school health programs nationwide. |
| 21 | Magis [21] | 2010 | Assess the relationship between social sustainability and community resilience. | Content analysis | Community resilience is the ability to thrive in an environment characterized by change. | Introduced the Community Resilience Self Assessment. |
| 22 | Norris et al. [22] | 2008 | To provide a comprehensive theoretical framework for understanding resilience, which incorporates the concepts of stress, adaptability, wellbeing, and resource dynamics. | Literature review. | Community resilience links adaptive capacities to adaptation after a disturbance. | Emphasizes the importance of reducing risk, engaging local people, and planning for unknowns. |
| 23 | Abas et al. [23] | 2020 | Explore Disaster Resilience Education (DRE) programmes in a secondary school in Kelantan. | Questionnaire-based survey. | Minimal DRE programmes conducted, but good awareness level among school community. | Findings can guide local authorities to enhance resilience programmes in schools. |
| 24 | Hooli et al. [24] | 2023 | Examine the impact of Service Learning on EFL teacher education students. | Qualitative study. | Service Learning facilitates the development of competencies in the realms of academia, professionalism, and physical welfare. | Highlights the benefits of Service Learning in university education. |
| 25 | Darling et al. [25] | 2021 | Determine whether or not primary schools require a Mental Health and Wellbeing Coordinator position and assess its efficacy and implementation. | Quasi-experimental cluster study. | Evaluating the conviction of educators in their ability to promote the mental health and well-being of students. | Major mental health service reform will be guided by the findings. |
| 26 | Nifa et al. [26] | 2017 | Explore the importance of disaster education in Malaysia. | Quantitative surveys | Highlights the significance of education in fostering disaster safety and resilience consciousness. | Highlights the need for a flood disaster education program in Malaysia. |
| 27 | Eley et al. [27] | 2013 | The correlation between personality traits and resilience in physicians was investigated. | Cross-sectional study. | Cooperation, maturity, responsibility, optimism, and perseverance are all personality traits that are linked to resilience. | Suggests strategies to enhance resilience by considering key personality traits. |
| 28 | Rezazadeh et al. [28] | 2023 | Conduct research on the elements that contribute to the resilience of Iranian bilingual EFL teachers in state and non-profit institutions. | Mixed-methods | The factors that contribute to the resilience of teachers in public and private institutions are not significantly different. | In non-profit institutions, emphasizes the significance of teacher development programs, student-teacher rapport, and classroom advancement. |
| 29 | Schelvis et al. [29] | 2014 | Explore the significance, nature, and various viewpoints regarding the resilience of educators, teams, and institutions. | Literature review, and survey. | Four abilities vital for developing resilience are anticipation, monitoring, responding, and learning. | The resilience perspective is promising for the educational sector. |
| 30 | Muñoz et al. [30] | 2020 | Examine disaster education and school safety in five different South American and Caribbean nations. | Case studies | Puerto Rico shows innovative examples of reflection on Hurricane Maria. Brazil encourages schools to become producers of knowledge. | Highlights the importance of school safety and disaster education in the region and the need for innovative approaches. |
| 31 | Sheridan et al. [31] | 2022 | An Analysis of the Implementation of Adaptability and Resilience among Early Career Teachers in Secondary Schools in Australia. | Semi-structured interviews | In response to instructional challenges, ECTs necessitate resilient cognitive, behavioral, and emotional adjustment. | Strategic, individualized, interdependent support networks within schools should be the focus of ECTs' professional development. |
| 32 | Goto et al. [32] | 2022 | Assess children's experiences of the “Creative Health” project in Fukushima. | Workshops; Questionnaires; Participants: 105 students aged 9–11 | Positive changes in students' perceptions of health and community. | The Creative Health project enhances children's creativity and autonomy in the community. |
| 33 | Brown and Westaway [33] | 2011 | Consolidate insights from diverse disciplines regarding agency, capacity, and resilience in order to comprehend environmental change. | Literature review | Subjective and relational factors must be taken into account alongside objective measures of capacity. | Distinguish among transformation, adaptation, and coping as reactions to stressors. |
| 34 | Li [34] | 2023 | Investigate the connections between exhaustion, resilience, self-efficacy, and emotion regulation among Chinese EFL instructors. | Self-report assessments; Confirmatory factor analysis; Structural equation modeling | Burnout is negatively correlated with both teacher self-efficacy and resilience. Through the mediation of resilience, emotion regulation indirectly impacts fatigue. | The significance of emotion regulation in fostering the well-being of educators is underscored, and potential strategies for focused interventions are proposed. |
| 35 | Mutch [35] | 2023 | Assess the contribution of educational institutions to recovery and disaster management in the Asia-Pacific area. | Decade-long research in 5 countries | Despite believing they are underprepared and underrecognized, schools play a crucial role in post-disaster environments. | Recommendations for better recognition and support of schools in disaster policy and planning. |
| 36 | Guo et al. [36] | 2020 | Examine the impact of negative emotions and resilience on the correlation between the level of support provided by educators and the mental well-being of adolescents. | Questionnaires survey with 1228 Chinese adolescents | Resilience, teacher support, and negative emotions are all associated with mental health. | By increasing help-seeking behavior, affect control, and goal planning, and decreasing depression, teacher support can enhance students' mental health. |
| 37 | Le Brocque et al. [37] | 2017 | Examine the trauma-related mental health resources that are accessible in educational institutions across Australia and New Zealand. | Review of resources, and relevant analysis | Implementing trauma-informed practices in educational institutions improves post-trauma outcomes for students. | In the aftermath of trauma, additional funding is required for training and resource distribution, as well as mental health prioritization. |
| 38 | Bikar et al. [38] | 2021 | Analyze the ways in which educators at the elementary level fostered resilience in students throughout and in the aftermath of the Ranau Earthquake. | Interviews with 16 primary school students and teachers | Teachers employed WhatsApp as a means to impart emotional support and monitor stress levels in order to bolster students' resilience during and after the earthquake. | By utilizing social media to aid students' resilience during and after traumatic experiences, one can be proactive. |
| 39 | Hascher et al. [39] | 2021 | Justify the correlation between the resilience of educators and their overall well-being. | Review of papers addressing teacher wellbeing and resilience | In an effort to clarify the connection between resilience and wellbeing, the Aligning Wellbeing and Resilience in Education (AWaRE) model was developed. | Resilience and wellbeing in educational contexts may be better defined using the AWaRE model. |
| 40 | Johnston et al. [40] | 2012 | Determine the extent to which community involvement mitigated anxiety and trauma in the aftermath of two earthquakes in New Zealand. | Semi-structured interviews, analysis of papers, reports, and articles | Effective recovery depends on how society organizes and coordinates resources to assist recovery. | Describes the efficacy of community involvement in mitigating trauma and anxiety caused by earthquakes. |
| 41 | Amri et al. [41] | 2022 | Examine how DRR education is being incorporated into Indonesian institutions. | Content, and expert opinion analysis | DRR program at the school level can be integrated with initiatives at the family and community levels. | Emphasizes the importance of DRR education integration in schools. |
| 42 | Drzewiecki et al. [42] | 2020 | Examine the relationship between St. Kitts and Nevis's resistance to natural catastrophes and educational achievement. | Questionnaires with 343 adult residents | Resilience to catastrophes caused by natural hazards is positively correlated with professional schooling. | Emphasizes the need to increase disaster preparedness education at all levels. |
| 43 | Theron et al. [43] | 2022 | Examine school engagement trajectories for South African adolescents in stressed environments. | Longitudinal study with 172 adolescents | The specification of school engagement trajectories was achieved through the development of the AWaRE model. | The AWaRE model can help understand school engagement in stressed environments. |
| 44 | Trethowan and Nursey [44] | 2015 | Review teacher-based support programs for child and adolescent recovery post-disaster in Victorian schools. | Overview of a two-phased approach to teacher training. Development of a manual and online training. | Development of Psychological First Aid and Mental Health First Aid for teachers. Introduction of online training, Skills for Psychological Recovery for Teachers. | Emphasizes the importance of teacher training in supporting children and adolescents’ post-disaster. Provides a structured approach to teacher-based support. |
| 45 | Rico [45] | 2019 | Examine the role of school personnel and administrators in disaster preparedness and building community resilience. | Quantitative approach | School personnel and administrators, especially those teaching Science, Social Studies, and Health, play a crucial role in disaster management. Participants were from Manila, a hotspot for natural hazards. | Highlights the importance of school-community collaboration in disaster preparedness. Emphasizes the need for specialized training for school personnel in disaster management. |
| 46 | Osofsky et al. [46] | 2018 | To outline the St. Bernard Parish Youth Program's creation and assess if the initiative was linked to a rise in self-efficacy and a decline in trauma symptoms. | Quantitative approach based on survey. | Students that took part in the YLP had far better self-efficacy scores than their non-participating counterparts. | Promising programs, like the YLP, highlight the value of concurrent and interactive family, community, and individual support systems to improve the healing process for both parties. |
| 47 | Campos [47] | 2020 | To investigate the multidisciplinary connections between architecture and education with the goal of fostering resilience based on the "educational" value of architecture. | Qualitative approach | Resilience in school complexes may be strengthened by appropriate physical settings, which can benefit people's learning, health, and general well-being. | When well-planned, architecture adds a "educational" value that enhances wellbeing and inspires learning, making it a crucial ally of resilience. |

**List of documents**

1. Kangas-Dick K, O’Shaughnessy E, O’Shaughnessy E, O’Shaughnessy E. Interventions that promote resilience among teachers: A systematic review of the literature. Int J Sch Educ Psychol. 2020;8: 131–146. doi:10.1080/21683603.2020.1734125

2. Johnson VA, Ronan KR, Johnston DM, Peace R. Improving the Impact and Implementation of Disaster Education: Programs for Children Through Theory-Based Evaluation. Risk Anal. 2016;36: 2120–2135. doi:10.1111/risa.12545

3. Cutter SL, Barnes L, Berry M, Burton C, Evans E, Tate E, et al. A place-based model for understanding community resilience to natural disasters. Glob Environ Chang. 2008;18: 598–606. doi:10.1016/j.gloenvcha.2008.07.013

4. Apronti P, Osamu S, Otsuki K, Kranjac-Berisavljevic G. Education for Disaster Risk Reduction (DRR): Linking Theory with Practice in Ghana’s Basic Schools. Sustainability. 2015;7: 9160–9186. doi:10.3390/su7079160

5. Mead T, Pietsch C, Matthew V, Lipkin-Moore S, Metzger E, Avdeev I V., et al. Leveraging a Community of Practice to Build Faculty Resilience and Support Innovations in Teaching during a Time of Crisis. Sustainability. 2021;13: 10172. doi:10.3390/su131810172

6. Zhu TT, Zhang YJ. An investigation of disaster education in elementary and secondary schools: evidence from China. Nat Hazards. 2017;89: 1009–1029. doi:10.1007/s11069-017-3004-2

7. Edmeade J, Buzinde CN. Educators’ personal resilience in the context of disasters triggered by natural hazards: The case of the United States Virgin Islands (USVI). Int J Disaster Risk Reduct. 2021;66: 102571. doi:10.1016/j.ijdrr.2021.102571

8. Wiwik Astuti NM, Werdhiana IK, Wahyono U. Impacts of direct disaster experience on teachers’ knowledge, attitudes and perceptions of disaster risk reduction curriculum implementation in Central Sulawesi, Indonesia. Int J Disaster Risk Reduct. 2021;53: 101992. doi:10.1016/j.ijdrr.2020.101992

9. Oktari RS, Shiwaku K, Munadi K, Syamsidik, Shaw R. Enhancing community resilience towards disaster: The contributing factors of school-community collaborative network in the tsunami affected area in Aceh. Int J Disaster Risk Reduct. 2018;29: 3–12. doi:10.1016/j.ijdrr.2017.07.009

10. Dufty N. Natural hazards education in Australian schools: How can we make it more effective? Aust J Emerg Manag. 2009;24: 13–16.

11. Seyle DC, Widyatmoko CS, Silver RC. Coping with natural disasters in Yogyakarta, Indonesia: A study of elementary school teachers. Sch Psychol Int. 2013;34: 387–404. doi:10.1177/0143034312446889

12. Codreanu TA, Celenza A, Jacobs I. Does disaster education of teenagers translate into better survival knowledge, knowledge of skills, and adaptive behavioral change? A systematic literature review. Prehosp Disaster Med. 2014;29: 629–642. doi:10.1017/S1049023X14001083

13. Sung-Chin Chung, Cherng-Jyh Yen. Disaster Prevention Literacy among School Administrators and Teachers: A Study on the Plan for Disaster Prevention and Campus Network Deployment and Experiment in Taiwan. J Life Sci. 2016;10: 203–214. doi:10.17265/1934-7391/2016.04.006

14. Gaillard JC, Mercer J. From knowledge to action: Bridging gaps in disaster risk reduction. Prog Hum Geogr. 2013;37: 93–114. doi:10.1177/0309132512446717

15. Cels J, Rossetto T, Little AW, Dias P. Tsunami preparedness within Sri Lanka’s education system. Int J Disaster Risk Reduct. 2023;84: 103473. doi:10.1016/j.ijdrr.2022.103473

16. Jarupongputtana C, Mangkhang C, Dibyamandala J, Manokarn M. Interdisciplinary Community Based Learning to Enhance Competence of Digital Citizenship of Social Studies Pre-Service Teacher’s in Thai Context: Pedagogical Approaches Perspective. J Curric Teach. 2022;11: 171–183. doi:10.5430/jct.v11n4p171

17. Johnson VA, Ronan KR, Johnston DM, Peace R. Implementing disaster preparedness education in New Zealand primary schools. Disaster Prev Manag An Int J. 2014;23: 370–380. doi:10.1108/DPM-09-2013-0151

18. Abrash Walton A, Marr J, Cahillane MJ, Bush K. Building Community Resilience to Disasters: A Review of Interventions to Improve and Measure Public Health Outcomes in the Northeastern United States. Sustainability. 2021;13: 11699. doi:10.3390/su132111699

19. Thayaparan M, Malalgoda C, Keraminiyage K, Amaratunga D. Disaster Management Education through Higher Education – Industry Collaboration in the Built Environment. Procedia Econ Financ. 2014;18: 651–658. doi:10.1016/s2212-5671(14)00987-3

20. Michael S, Dittus P, Epstein J. Family and community involvement in schools: Results from the school health policies and programs study 2006. J Sch Health. 2007;77: 567–587. doi:10.1111/j.1746-1561.2007.00236.x

21. Magis K. Community resilience: An indicator of social sustainability. Soc Nat Resour. 2010;23: 401–416. doi:10.1080/08941920903305674

22. Norris FH, Stevens SP, Pfefferbaum B, Wyche KF, Pfefferbaum RL. Community resilience as a metaphor, theory, set of capacities, and strategy for disaster readiness. Am J Community Psychol. 2008;41: 127–150. doi:10.1007/s10464-007-9156-6

23. Abas MA, Ibrahim NE, Wee ST, Sibly S, Mohamed S. Disaster Resilience Education (DRE) Programmes in Schools: A Case Study in Kelantan, Malaysia. IOP Conf Ser Earth Environ Sci. 2020;549. doi:10.1088/1755-1315/549/1/012078

24. Hooli EM, Corral-Robles S, Ortega-Martín JL, Baena-Extremera A, Ruiz-Montero PJ. The Impact of Service Learning on Academic, Professional and Physical Wellbeing Competences of EFL Teacher Education Students. Int J Environ Res Public Health. 2023;20. doi:10.3390/ijerph20064852

25. Darling S, Dawson G, Quach J, Smith R, Perkins A, Connolly A, et al. Mental health and wellbeing coordinators in primary schools to support student mental health: protocol for a quasi-experimental cluster study. BMC Public Health. 2021;21: 1–14. doi:10.1186/s12889-021-11467-4

26. Nifa FAA, Abbas SR, Lin CK, Othman SN. Developing a disaster education program for community safety and resilience: The preliminary phase. AIP Conference Proceedings. 2017. p. 020005. doi:10.1063/1.5005338

27. Eley DS, Robert Cloninger C, Walters L, Laurence C, Synnott R, Wilkinson D. The relationship between resilience and personality traits in doctors: Implications for enhancing well being. PeerJ. 2013;2013: 1–16. doi:10.7717/peerj.216

28. Rezazadeh K, Janebi Enayat M, Poorebrahim F. Exploring bilingual EFL teacher resilience in the Iranian non-profit and state schools: a mixed-methods study. Asian-Pacific J Second Foreign Lang Educ. 2023;8. doi:10.1186/s40862-023-00196-3

29. Schelvis RMC, Zwetsloot GIJM, Bos EH, Wiezer NM. Exploring teacher and school resilience as a new perspective to solve persistent problems in the educational sector. Teach Teach Theory Pract. 2014;20: 622–637. doi:10.1080/13540602.2014.937962

30. Muñoz VA, Carby B, Abella EC, Cardona OD, López-Marrero T, Marchezini V, et al. Success, innovation and challenge: School safety and disaster education in South America and the Caribbean. Int J Disaster Risk Reduct. 2020;44. doi:10.1016/j.ijdrr.2019.101395

31. Sheridan L, Andersen P, Patulny R, McKenzie J, Kinghorn G, Middleton R. Early career teachers’ adaptability and resilience in the socio-relational context of Australian schools. Int J Educ Res. 2022;115: 102051. doi:10.1016/j.ijer.2022.102051

32. Goto A, Williams AL, Okabe S, Koyama Y, Koriyama C, Murakami M, et al. Empowering Children as Agents of Change to Foster Resilience in Community: Implementing “Creative Health” in Primary Schools after the Fukushima Nuclear Disaster. Int J Environ Res Public Health. 2022;19. doi:10.3390/ijerph19063417

33. Brown K, Westaway E. Agency, capacity, and resilience to environmental change: Lessons from human development, well-being, and disasters. Annu Rev Environ Resour. 2011;36: 321–342. doi:10.1146/annurev-environ-052610-092905

34. Li S. The effect of teacher self-efficacy, teacher resilience, and emotion regulation on teacher burnout: a mediation model. Front Psychol. 2023;14: 1–13. doi:10.3389/fpsyg.2023.1185079

35. Mutch C. How schools build community resilience capacity and social capital in disaster preparedness, response and recovery. Int J Disaster Risk Reduct. 2023;92: 103735. doi:10.1016/j.ijdrr.2023.103735

36. Guo J, Liu L, Zhao B, Wang D. Teacher Support and Mental Well-Being in Chinese Adolescents: The Mediating Role of Negative Emotions and Resilience. Front Psychol. 2020;10: 1–11. doi:10.3389/fpsyg.2019.03081

37. Le Brocque R, De Young A, Montague G, Pocock S, March S, Triggell N, et al. Schools and Natural Disaster Recovery: The Unique and Vital Role That Teachers and Education Professionals Play in Ensuring the Mental Health of Students Following Natural Disasters. J Psychol Couns Sch. 2017;27: 1–23. doi:10.1017/jgc.2016.17

38. Bikar SS, Rathakrishnan B, Kamaluddin MR, Che Mohd Nasir N, Mohd Nasir MA. Social Sustainability of Post-Disaster: How Teachers Enable Primary School Students to Be Resilient in Times of Ranau Earthquake. Sustainability. 2021;13: 7308. doi:10.3390/su13137308

39. Hascher T, Beltman S, Mansfield C. Teacher wellbeing and resilience: towards an integrative model. Educ Res. 2021;63: 416–439. doi:10.1080/00131881.2021.1980416

40. Johnston D, Becker J, Paton D. Multi-agency community engagement during disaster recovery: Lessons from two New Zealand earthquake events. Disaster Prev Manag. 2012;21: 252–268. doi:10.1108/09653561211220034

41. Amri A, Lassa JA, Tebe Y, Hanifa NR, Kumar J, Sagala S. Pathways to Disaster Risk Reduction Education integration in schools: Insights from SPAB evaluation in Indonesia. Int J Disaster Risk Reduct. 2022;73: 102860. doi:10.1016/j.ijdrr.2022.102860

42. Drzewiecki DM, Wavering HM, Milbrath GR, Freeman VL, Lin JY. The association between educational attainment and resilience to natural hazard-induced disasters in the West Indies: St. Kitts & Nevis. Int J Disaster Risk Reduct. 2020;47: 101637. doi:10.1016/j.ijdrr.2020.101637

43. Theron L, Ungar M, Höltge J. Pathways of resilience: Predicting school engagement trajectories for South African adolescents living in a stressed environment. Contemp Educ Psychol. 2022;69: 102062. doi:10.1016/j.cedpsych.2022.102062

44. Trethowan V, Nursey J. Helping children and adolescents recover from disaster: A review of teacher-based support programs in Victorian schools. Aust J Emerg Manag. 2015;30: 17–20.

45. Rico GCS. School-Community Collaboration: Disaster Preparedness Towards Building Resilient Communities. Int J Disaster Risk Manag. 2019;1: 45–59. doi:10.18485/ijdrm.2019.1.2.4

46. Osofsky H, Osofsky J, Hansel T, Lawrason B, Speier A. Building resilience after disasters through the youth leadership program: The importance of community and academic partnerships on youth outcomes. Prog Community Heal Partnerships Res Educ Action. 2018;12: 3–4. doi:10.1353/cpr.2018.0013

47. Campos P. Resilience, education and architecture: The proactive and “educational” dimensions of the spaces of formation. Int J Disaster Risk Reduct. 2020;43: 101391. doi:10.1016/j.ijdrr.2019.101391
